# Facilitating Corticomotor Excitability of the Contralesional Hemisphere Using Non-Invasive Brain Stimulation to Improve Upper Limb Motor Recovery from Stroke—A Scoping Review

**DOI:** 10.3390/jcm13154420

**Published:** 2024-07-28

**Authors:** Pui Kit Tam, Nicodemus Edrick Oey, Ning Tang, Guhan Ramamurthy, Effie Chew

**Affiliations:** 1 Division of Rehabilitation Medicine, Department of Medicine, National University Hospital, Singapore 119228, Singapore; mdctpk@nus.edu.sg (P.K.T.); nico.oey@nus.edu.sg (N.E.O.); ning_tang@nuhs.edu.sg (N.T.); 2 Yong Loo Lin School of Medicine, National University of Singapore, Singapore 117549, Singapore; 3 BG Institute of Neurosciences, BG Hospital, Tiruchendur, Tuticorin 628216, Tamil Nadu, India; guhanramamurthy@gmail.com

**Keywords:** intracerebral hemorrhage, stroke, non-invasive brain stimulation, upper limb impairment

## Abstract

Upper limb weakness following stroke poses a significant global psychosocial and economic burden. Non-invasive brain stimulation (NIBS) is a potential adjunctive treatment in rehabilitation. However, traditional approaches to rebalance interhemispheric inhibition may not be effective for all patients. The supportive role of the contralesional hemisphere in recovery of upper limb motor function has been supported by animal and clinical studies, particularly for those with severe strokes. This review aims to provide an overview of the facilitation role of the contralesional hemisphere for post-stroke motor recovery. While more studies are required to predict responses and inform the choice of NIBS approach, contralesional facilitation may offer new hope for patients in whom traditional rehabilitation and NIBS approaches have failed.

## 1. Introduction

Stroke (ischemic and hemorrhagic) is the third leading cause of disability worldwide, accounting for significant disability-adjusted life years lost [1,2] and an ever-increasing medical cost, which is projected to be 183 billion dollars by 2030 in the United States alone [3]. A major contributor to disability lies in the persistence of upper limb motor impairment in more than half of all stroke patients [4], which reduces the ability to perform activities of daily living and greatly worsens quality of life [5] despite various treatments and rehabilitation techniques for upper extremity motor recovery [6]. Non-invasive brain stimulation (NIBS) including repetitive transcranial magnetic stimulation (rTMS) and transcranial direct current stimulation (tDCS) have been investigated as potential effective adjuncts to conventional rehabilitation [7]. NIBS has been studied for use as an adjunctive therapeutic modality in various post-stroke conditions, such as motor impairment, aphasia, neglect, cognitive dysfunction, and central pain [8,9,10]. Physiologically, anodal tDCS and high-frequency rTMS (HF-rTMS) cause facilitation of cortical excitability, whereas cathodal tDCS and low-frequency rTMS (LF-rTMS) induce intracortical inhibition, with neurobiological aftereffects persisting beyond the duration of the stimulation [11]. Unfortunately, the evidence supporting the use of rTMS and tDCS in post-stroke motor rehabilitation is equivocal: Meta-analyses suggest possible benefits, but a recent large multicenter, randomized, controlled trial failed to demonstrate superiority of rTMS over sham stimulation [12]. Currently, both tDCS and rTMS are still not approved for use in upper limb rehabilitation by the Food and Drug Administration of the United States, although in some parts of the world, they are used off-label or on a compassionate basis [13,14].

One important factor is that trials to date have not adequately considered residual reserves and capacity for recovery when selecting neuromodulatory protocols [15]. A differential effect of HF-rTMS over the ipsilesional motor cortex (iM1) to improve hand motor function has been noted, with effect seen only for those with subcortical but not cortical lesions [16]. Even with invasive motor cortex stimulation, where iM1 was identified by fMRI for implantation of stimulatory epidural electrodes, corticospinal tract (CST) integrity evidenced by movement on stimulation prior to intervention portended better outcomes [17]. These studies suggest the need for stratification with consideration of the integrity of the stimulated tracts.

Traditional approaches to NIBS for post-stroke functional recovery are based on the premise of rebalancing interhemispheric inhibition [18,19]. Pathologically increased inhibition of the ipsilesional hemisphere by the contralesional hemisphere is thought to impede functional recovery of the affected hemisphere [20]. Hence, most NIBS protocols for motor recovery seek to increase the excitability of the ipsilesional motor cortex (iM1) using HF-rTMS, anodal tDCS, or intermittent theta-burst stimulation (TBS) or to inhibit the contralesional motor cortex (cM1) using low-frequency rTMS (LF-rTMS), cathodal tDCS, or continuous TBS, with the aim to restore interhemispheric symmetry [21]. However, this model may not be possible with inadequate functional reserves, e.g., in more extensive injuries [22,23,24].

For patients with low functional reserves, an alternative vicariation model was proposed, where facilitation of alternative pathways may be more effective in restoring function [15]. In a novel take on the bimodal balance recovery model, Plow et al. adopted a tailored approach, in which patients with good structural reserve were treated with the “traditional” NIBS approaches, while patients with reduced CST integrity (defined by fractional anisotropy [FA] of <0.5 on diffusion tensor imaging) were found to potentially benefit more from facilitatory-rTMS to the contralesional hemisphere [25]. For motor impairments, possible targets for neuromodulation include facilitation of the contralesional primary motor cortex and facilitation or inhibition of the contralesional premotor cortices and supplementary motor areas.

Multiple lines of evidence support the facilitatory role of the contralesional hemisphere in motor recovery. In rat stroke models, structural and functional crossing of contralesional corticospinal neurons and motor remapping are associated with behavioral recovery [26,27], and greater contralesional premotor activity is correlated with larger stroke lesions [28]. Similarly, functional MRI studies in stroke patients show that secondary motor areas of the contralesional hemisphere are recruited in patients with more severe damage [29,30], and electrophysiological studies show ipsilateral activation being unmasked as latent pathways [31].

Facilitation of contralesional pathways was proposed as a possible approach for tailored stimulation, especially in poorly recovered severe stroke patients [32]. However, randomized clinical trials investigating contralesional facilitation are limited. A recent systematic review found that 68 out of the 70 clinical studies using rTMS for post-stroke upper limb motor recovery used the rebalancing of interhemispheric inhibition (IHI) model, while only 2 studies investigated facilitation of the contralesional hemisphere [33]. 

In this scoping review, we aim to assess the efficacy of facilitatory NIBS to the contralesional hemisphere to improve motor outcomes of the paretic upper limb in stroke survivors. Secondarily, we aimed to assess the neurophysiological changes and proposed biomarkers for response to this stimulation paradigm.

## 2. Methods

### 2.1. Study Design

A scoping review was conducted according to the PRISMA (Preferred Reporting Items for Systematic Reviews and Meta-Analyses) standards [34]. 

### 2.2. Search Strategy and Selection Criteria

**Figure 1:**
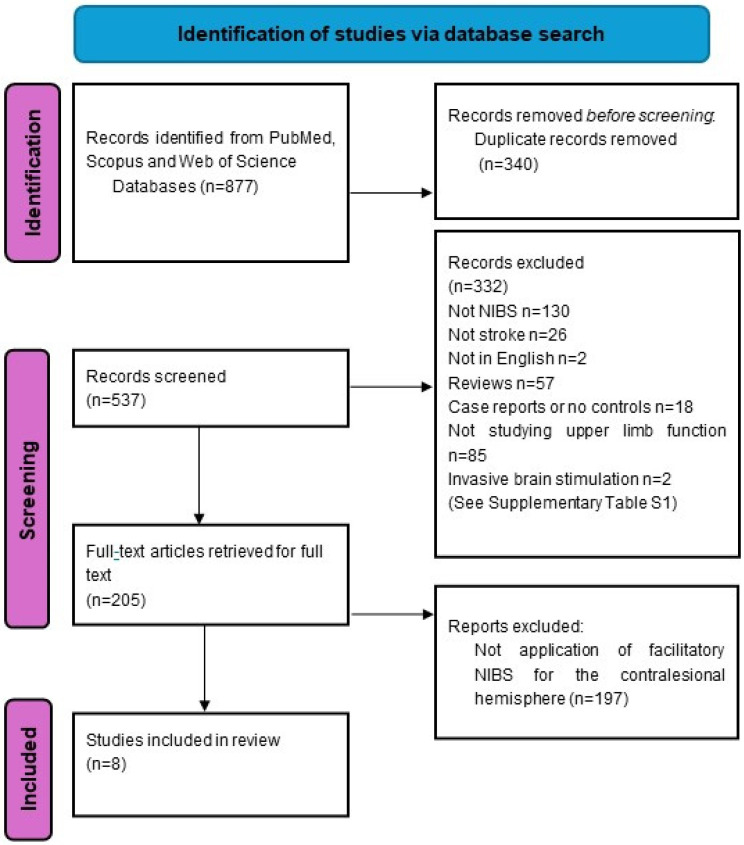
Flowchart detailing systematic review process.

We searched three databases, namely PubMed, Web of Science, and Scopus, from inception to 22 December 2023 (Figure 1). The following keywords were used: “stroke”, “cerebrovascular accident”, “non-invasive brain stimulation”, “NIBS”, “transcranial direct current stimulation”, “tDCS”, “repetitive transcranial magnetic stimulation”, “rTMS’, “theta burst stimulation”, “TBS”, “motor”, “contralesional”, “unaffected”, “non-lesioned”, “ipsilateral”, “stimulatory”, “facilitatory”, “facilitation”, “anodal”, “intermittent”, and “high frequency”. The detailed search strategy is attached in Appendix A.

We identified eight studies from the above search strategy related to the application of NIBS to the contralesional hemisphere for improvement of upper limb motor function in stroke (Table 1). We did not specifically search for the term “upper limb” or “upper extremity”, as these search criteria highly limited the pool of potential studies, and we opted instead to manually screen for all studies to maximize yield (Figure 1, Supplementary Table S1). Studies were also excluded if they were not published in English, did not involve facilitatory non-invasive brain stimulation to the contralesional hemisphere, did not include a control stimulation experiment or group, included outcome measures that did not include upper limb motor function, did not involve any human subjects with stroke, or were case reports, study protocols, reviews, or meta-analyses.

## 3. Results

The search and selection process was shown in Figure 1. (For the detailed list and reasons for exclusion, please see Supplementary Table S1). Eight studies were included in this review, and they are summarized in Table 1. Seven out of the eight studies included only adults with stroke, while one included children and young adults with perinatal stroke or other non-traumatic brain injuries. Three studies used high-frequency rTMS (HF-rTMS) as the NIBS modality, while four used anodal tDCS. The remaining study is a combination of tDCS to cM1 plus HF-rTMS to iM1.

### 3.1. Study Design and Study Quality

In this review, we identified one randomized, controlled trial with repeated sessions of NIBS being applied as a therapeutic intervention over two weeks [35] (Table 1). In this study, the sample size was small, and there was an assessment immediately post intervention but no longer-term outcomes. The rest of the identified studies constituted proof-of-concept studies with crossover study design, where 1–2 sessions of contralesional NIBS were compared with sham or other NIBS protocol. None of the studies reported long-term outcome measures beyond 6 months.

### 3.2. Stroke Timing, Type, and Lesion Location

Six out of eight studies recruited only chronic stroke patients (at least three months post stroke). One study recruited only subacute cases (within three months of stroke onset), while the remaining study recruited participants ranging from one to forty-eight months post stroke. Six studies recruited both ischemic and hemorrhagic strokes, while the remaining two did not specify the stroke type. Three studies recruited patients with lesions in all locations (cortical, subcortical, and brainstem), another three only included cortical or subcortical lesions, and two did not specify the site.

### 3.3. Types of NIBS Interventions and Sites of Stimulation

The NIBS protocols and sites of application were diverse. Three studies applied HF-rTMS to the contralesional hemisphere: One study applied it to cM1 and compared it with LF-rTMS to cM1 or sham rTMS [36]. The second study applied LF-rTMS to cM1, HF-rTMS to contralesional premotor cortex (cPMD), and sham rTMS in a randomized sequence, and in a subgroup of participants with unmeasurable motor evoked potential (MEP), application of HF-rTMS to cM1 and cPMD was compared [37]. The third rTMS study compared HF-rTMS to cPMD and iM1 in a crossover design [38].

Four studies used tDCS on the contralesional hemisphere: One study compared the effects of a single session of anodal tDCS to cM1 with cathodal tDCS to cM1 or sham tDCS in a randomized, crossover design [39]. The second study compared anodal tDCS to cM1 with sham tDCS [40]. The third study recruited subjects with perinatal stroke or non-traumatic brain injury and applied tDCS according to the presence or absence of MEP in the paretic upper limb, where subjects with measurable MEP had anodal tDCS applied to iM1, while those without measurable MEP received anodal tDCS to cM1 [41]. The fourth study compared the effects of a single session of anodal tDCS to iM1 and cM1 or cathodal tDCS to cM1 in a randomized order [42]. The remaining study tested the effects of applying either anodal or cathodal tDCS to cM1 in combination with HF-rTMS to iM1 [43].

### 3.4. Neurophysiological and Behavioral Outcome Measures

Seven studies measured electrophysiological responses to NIBS and the associated behavioral outcomes: Six of them measured corticomotor excitability by recording MEP responses from both hemispheres. One study measured reciprocal inhibition by comparing the H-reflex of the wrist flexor and wrist extensor of the affected upper limb [40]. Electromyographic activity of upper limb muscles was measured in two studies [36,39]. The cortical silent period was measured in two studies [37,38].

A variety of behavioral outcomes were used, including measurements of accuracy and speed while performing a sequential finger-tapping task [43], upper limb reaching tasks [37,42], and a bimanual force coordination test as measured during an elbow flexion movement [38]. Only one study reported changes in the ability to perform activities of daily living (the Fugl–Meyer Assessment [FMA] and the Barthel Index [BI]) before and after 14 sessions of HF-rTMS [36].

Of the six studies that measured MEP, five reported an increase in contralesional corticomotor excitability (Table 1) [36,37,38,39,41]. Ipsilesional corticomotor excitability was more variable, as some of the participants had no measurable MEP. Three studies compared the neurophysiological effect of a single session of anodal tDCS to cM1 with sham tDCS in a randomized, crossover design [39,42,43]. Klomjai et al. investigated the effect of tDCS on reciprocal inhibition (RI, as measured by H-reflex of reciprocal muscle group, flexor carpi radialis, and extensor carpi radialis) of the paretic hand [40]. RI is mediated by spinal neuron networks controlling antagonistic muscle activation with inputs from bilateral descending reticulospinal tracts. Anodal tDCS decreased the RI of the paretic and non-paretic arm relative to sham in stroke participants as compared to healthy controls, who had increased RI in the contralateral arm with tDCS. In addition, greater severity of upper limb paresis correlated with greater suppression of RI with tDCS. The authors concluded that the effect of contralesional tDCS on paretic RI may be mediated by uncrossed or indirect pathways such as the cortico-reticulospinal tracts [40]. 

### 3.5. Studies Investigating the Facilitation of Contralesional Primary Motor Cortex

Of the six studies that investigated facilitation of cM1 (Table 1), five reported behavioral outcomes. Of these, one randomized, controlled trial with repeated sessions over 14 days reported significant functional improvement (all participants were MEP-negative) [36], one reported a trend towards better performance (statistically not significant) [39], two reported no benefit [42,43], and one did not differentiate between facilitation of cM1 and iM1 [41].

McCambridge et al. measured MEP with ipsilesional and contralesional TMS and electromyography (EMG) of the paretic arm in 10 chronic stroke participants during a circling task after single sessions of anodal, cathodal, or sham tDCS [39]. Contralateral paretic MEPs improved after anodal tDCS but not cathodal or sham, as did contralateral non-paretic MEPs. There was also a trend of less suppression of the late cortical component of the paretic arm EMG as well as improved performance of a circle-drawing task after contralesional anodal tDCS, which did not correlate with changes in paretic MEP. Hence, it was suggested that the effect of contralesional anodal tDCS may be mediated through crossed and uncrossed pathways, including cortico-reticular projections onto cervical propriospinal neurons innervating the paretic limb. 

Nemanich applied a stratified approach to tDCS in children and young adults with perinatal stroke or other non-traumatic brain injuries [41]. Anodal tDCS was applied to iM1 for one session with motor training if MEP was measurable and to the cM1 if MEP was absent. Participants were randomized to real or sham tDCS. More participants who received real tDCS had increases in MEP amplitude, although this result was not statistically significant, and more participants in the sham group were MEP-negative at baseline. The authors did not separately report the responses of the MEP-negative and -positive groups and the associated behavioral changes. 

Kwon et al. investigated preconditioning vs. simultaneous tDCS in chronic stroke with the following protocols in randomized order: (i) anodal tDCS to cM1 with simultaneous HF-rTMS to iM1; (ii) cathodal tDCS to cM1 with simultaneous HF-rTMS to iM1; (iii) anodal tDCS to cM1 preconditioning followed by HF-rTMS to iM1; (iv) cathodal tDCS to cM1 preconditioning followed by HF-rTMS to iM1; and (v) sham tDCS with HF-rTMS to iM1 [43]. Simultaneous cathodal tDCS to cM1 and HF-rTMS to iM1 resulted in better motor improvement and corticomotor excitability. 

Yao et al. applied tDCS (anodal tDCS to iM1 and cM1 or cathodal tDCS to cM1 in a randomized order) prior to a reaching task of the affected upper limb using a robotic device [42]. The outcome measured was maximal shoulder abduction torques and reaching distance. Anodal tDCS to cM1 had no effect on the reaching tasks, while cathodal tDCS to cM1 decreased the reaching distance. Anodal tDCS to iM1 tended to increase reaching distance, though it was not statistically significant. The subjects recruited had Fugl–Meyer Assessment Upper Extremity (FMA-UE) scores ranging from 20 to 40, but there was no analysis according to the baseline FMA-UE score.

Wang et al. conducted a randomized, controlled trial evaluating the effect of repeated facilitatory NIBS to cM1 [36]. Forty-five chronic, severe stroke survivors were randomized to receive 14 sessions of either HF, LF, or sham rTMS of cM1. All subjects were without measurable MEP from the paretic arm on TMS of the iM1. HF rTMS resulted in significantly greater improvement compared to LF and sham rTMS in terms of motor performance, contralesional cortical excitability, conductivity, and EMG activation of key muscles in the affected limbs. Improvement in motor performance correlated with decreased contralesional MEP latency, indicating improved contralesional conductivity. By contrast, Kwon et al., who studied patients with milder stroke (FMA 50.7 ± 8.5; MEP-positive), found that cathodal tDCS to cM1 combined with HF-rTMS to iM1 improved motor performance compared to anodal tDCS to cM1 combined with HF-rTMS to iM1.

### 3.6. Studies Investigating the Facilitation of Contralesional Dorsal Premotor Cortex

In the study by Sankarasubramanian et al., participants received a session of LF-rTMS to cM1, HF rTMS to cPMD, and sham rTMS in a randomized sequence [37]. Using reductions in the reaction time to perform proximal paretic limb reaching as a measure of improved upper limb function, participants with more severe upper limb impairment (lower score of the proximal subscore of FMA-upper extremity [FMA-UE]) and more severe damage to the corticospinal tract (lower score of fractional anisotropy [FA] of posterior limb of internal capsule on MRI) had greater improvement in performance with facilitation of cPMD than with inhibition of cM1 or facilitation of cM1 or facilitation of iPMD. In contrast, less impaired participants had greater improvement with LF-rTMS of cM1 compared to facilitation of cPMD. Paretic MEP was not increased after HF-rTMS to cPMD, although non-paretic MEP was increased. The ipsilateral silent period (iSP), reflecting interhemispheric inhibition on iM1, was decreased after cPMD facilitation but not cM1 facilitation and was correlated with improvement in reaching time. 

Liao et al. compared the effects of HF-rTMS to cPMD versus iM1 on a bimanual coordination task in a crossover study [38]. Participants with less severe upper limb impairment and stronger interhemispheric inhibition (higher iSP) showed improved bimanual coordination with iM1 stimulation. Increased ipsilesional excitability and decreased iSP were also seen. Those with lower FMA-UE scores and lower iSP had better motor improvement with cPMD facilitation, associated with a reduction in iSP. For both cPMD and iM1 responders, response was associated with an increase in ipsilesional cortical excitability and a decrease in intracortical inhibition in the lesioned cortex (as measured by the contralateral silent period), while IHI to the lesioned cortex (as measured by iSP inhibition) decreased for cPMD responders but not for iM1 responders, suggesting that cPMD rTMS-induced enhancement of function may be through transcallosal pathways. 

**Table 1:** A summary of the eight identified studies investigating contralesional NIBS in this review.

Studies Investigating the Facilitation of cM1									
	Study Author, Year (n)	NIBS Type	MEP recorded	Study design/Number of sessions	Stimulation sites	Stimulation protocols	Severity of upper limb impairment Stratification	Stroke characteristics	Summary of Key Results
1	Wang et al., 2020 [36] (n = 45)	rTMS	APB and BB of non-paretic hand	Randomized, single-blinded; daily sessions for 14 days	cM1	10 Hz rTMS at 100% RMT to cM1 (n = 15) 1 Hz rTMS at 100% RMT to cM1 (n = 15) Sham rTMS by coil rotation 90° (n = 15)	Severe: -Total FMA 8-38/100 (all subjects were MEP-) No stratification	Chronicity: Subacute (2 weeks–3 months post stroke) Type: Ischemic or hemorrhagic; cortical or subcortical	Better improvement in HF group in FMA, BI, contralesional cortical excitability, conductivity, and RMS-SEMG of the key muscles. No difference in LF group vs. sham. Contralesional cortical conductivity correlated with motor recovery.
2	Kwon et al., 2016 [43] (n = 20)	tDCS + rTMS	FDI of the non-paretic hand	Randomized, cross-over; single session ×5	cM1, iM1 bihemispheric concurrent stimulation	Anodal 2 mA tDCS cM1 + 10 Hz rTMS at 90% RMT iM1 Cathodal tDCS cM1 + 10 Hz rTMS iM1; Anodal tDCS cM1, then 10 Hz rTMS iM1 Cathodal tDCS cM1, then 10 Hz rTMS iM1 Sham tDCS cM1 + 10 Hz rTMS iM1	Mild: all subjects MEP+ and FMA-UE range 39–65 No stratification	Chronicity: Chronic (>3 months post stroke) Type: Ischemic or hemorrhagic; cortical, subcortical, or brainstem	Anodal tDCS to cM1 during or before HF rTMS to iM1 did not improve performance of a sequential finger task (accuracy and time) or iM1 MEP amplitude. Concurrent cathodal tDCS over cM1 and HF rTMS of iM1 improved motor performance and iM1 MEP.
3	Nemanich et al., 2023 [41] (n = 14)	tDCS	APB and ED of both paretic and non-paretic hand	Randomized; single session over 1 h	MEP+→iM1 MEP-→cM1	Anodal tDCS 1.5 mA cM1 (n = 4) Anodal 1.5 mA tDCS iM1 (n = 4) Sham tDCS to iM1 (n = 2) Sham tDCS cM1 (n = 4)	Mixed: 42.8% MEP+ Stratification based on MEP status	Chronicity Chronic (13–14 years) Type: Both ischemic or hemorrhagic; location not specified	More subjects in real tDCS group had increased MEP amplitude compared to sham tDCS; however, the effect was not significant after adjusting for age.
4	McCambridge et al., 2018 [39] (n = 10)	tDCS	BB and ECR	Crossover, double-blinded; single sessions ×3	cM1	Anodal tDCS at 1 mA to cM1 (applied for 15 min) Cathodal tDCS at 1 mA to cM1 (applied for 15 min) Sham tDCS cM1	Mild-severe: FMA-UE 9-58; 40% subjects MEP- No stratification	Chronicity Chronic (>6 months post stroke) Type: Cortical or subcortical stroke; ischemic or hemorrhagic not specified	Anodal tDCS to cM1 increased both ipsi- and contralateral MEPs of the paretic arm, while cathodal tDCS had no effect. Trend towards improved paretic intralimb coordination as evidenced by improved circle drawing.
5	Klomjai et al., 2022 [40] (n = 21)	tDCS	-	Crossover; single sessions × 3	cM1	Anodal tDCS at 1.75 mA to cM1 (applied for 20 min) Sham tDCS cM1 (applied for 2 min)	Mild to moderate MEP not reported No stratification	Chronicity: Mixed (1–48 months) Type: Ischemic or hemorrhagic	Anodal tDCS reduced the reciprocal inhibition of flexor carpi radialis of the affected arm.
6	Yao et al., 2015 [42] (n = 9)	tDCS	-	Crossover; single sessions ×3	iM1 or cM1	Anodal tDCS at 0.8 mA to cM1 (15 min) Cathodal tDCS at 0.8 mA to cM1 (15 min) Anodal tDCS at 0.8 mA to iM1 (15 min) Sham tDCS	FMA-UE range 20–40 No stratification	Chronicity: Chronic Type: Not specified	Cathodal tDCS to cM1 decreased shoulder reaching distance of the affected side. Anodal tDCS to iM1 increased reaching (statistically not significant). Anodal tDCS to cM1 had no effect on reaching.
**Studies Investigating the Facilitation of cPMD**									
7	Sankarasubramanian et al., 2017 [37] (n = 15)	rTMS	EDC in non-paretic hand	Crossover; single sessions	cM1, cPMD and iPMD	1 Hz rTMS at 90% AMT to cM1 5 Hz rTMS at 90% AMT to cM1 5 Hz rTMS at 90% AMT to cPMD 5 Hz rTMS at 90% AMT to iPMD Sham rTMS over the same site (iM1)	Mild-severe: FMA-UE 7-63; 60% MEP+ and 40% MEP- Stratification by FMA and mean FA	Chronicity: Chronic (mean of 55 months post stroke) Type: Ischemic or hemorrhagic; cortical, subcortical, or brainstem	Those with low FMA-UE and low mean FA on MRI had greater improvement in paretic arm reaching time with HF rTMS to cPMD compared to LF rTMS to cM1. Facilitation of cPMD but not cM1 improved reaching time with reduction in iSP to the lesioned cortex. A decision tree for stratifying responders was proposed.
8	Liao et al., 2019 [38] (n = 14)	rTMS	BB in paretic and non-paretic upper limb	Cross-over; two sessions each ×2	cPMD iM1	5 Hz rTMS at 70% AMT to cPMD 5 Hz rTMS at 90% AMT to iM1	Mild-moderate: FMA-UE 20-65 and MEP+ Stratification based on FMA, ipsilateral silent period, and MEP ratio after rTMS	Chronicity: Chronic (>1 year post stroke) Type: Ischemic or hemorrhagic; cortical, subcortical, or brainstem	cPMD responders (n = 4) (improved interlimb force coordination and intermuscular coherence) had lower FMA-UE and higher iSP inhibition to the affected arm. iM1 responders (n = 10) had higher FMA-UE and lower iSP inhibition. cPMD responders showed a decrease in iSP inhibition with rTMS.

Abbreviations used: FMA (Fugl–Meyer Assessment); BI (Barthel Index); RMS-SEMG (root mean square of surface electromyography); FA (fractional anisotropy); MEP- (MEP absent in key muscles of stroke-affected arm on TMS of the lesioned primary motor cortex); MEP+ (MEP present in key muscles of stroke-affected arm on TMS of the lesioned primary motor cortex); HF (high frequency); AMT (active motor threshold); RMT (resting motor threshold); iM1 (ipsilesional primary motor cortex); cM1 (contralesional primary motor cortex); cPMD (contralesional dorsal premotor cortex); iPMD (ipsilesional dorsal premotor cortex); iSP (ipsilateral silent period); APB (abductor pollicis brevis); ED (extensor digitorum); EDC (extensor digitorum communis); FDI (flexor digitorum of index finger).

## 4. Discussion

To the best of our knowledge, this is the first scoping review of facilitatory NIBS to the contralesional hemisphere for upper limb motor recovery post stroke. The eight identified studies demonstrated that facilitation of the contralesional hemisphere was associated with increased contralesional corticomotor excitability, with variable effect on ipsilesional cortical excitability and transcallosal pathways and possible effect on spinal neuron networks. There is preliminary suggestion of greater motor improvement with contralesional facilitation as compared to facilitation of iM1 or inhibition of cM1 for those with severe motor impairments [37,38,40,43].

### 4.1. The Role of cM1 and cPMD in Stroke Recovery

Better understanding and characterization of post-stroke changes in interhemispheric interactions would greatly facilitate our choice of NIBS approach. However, contralesional influences on stroke motor recovery are still not fully understood. In healthy individuals, unilateral hand tasks involving higher accuracy or complexity activate ipsilateral M1 and the premotor cortex, with the degree of activation being related to the demand for accuracy [44]. This is in contrast to simple motor tasks (e.g., finger tapping), which recruit contralateral M1 and not the premotor cortices.

Soon after a stroke, decreased iM1 excitability is accompanied by increased bilateral recruitment of secondary motor networks with unilateral motor tasks involving the stroke-affected upper limb, including the contralesional sensorimotor, dorsal and ventral premotor cortex, supplementary motor areas, and anterior intraparietal sulcus [44,45,46,47,48]. With motor recovery, those with mild deficits show decreased contralesional activation over time to levels approaching healthy individuals [45,46,49,50]. In contrast, those with greater corticospinal damage show decreased intracortical inhibition of the contralesional motor cortex at rest [51] and increased activation in the cPMD, bilateral ventrolateral premotor cortices, and contralesional cerebellum with unilateral motor tasks [29,30,52,53]. The extent of CST damage correlated with the extent of bilateral activation of secondary motor networks and poorer outcomes [29,30]. Indeed, disruption of both cPMD and cM1 with TMS has been shown to slow reaction time and impair the quality of movement [35,54,55]. The degree of impairment was correlated to MRI lateralization to cPMD. Time post stroke also determines the contribution of the contralesional hemisphere such that the contralesional intraparietal sulcus exerted a detrimental effect post stroke, while cPMD may develop a supportive role later post stroke [56].

Lin et al. further found a differential influence of IHI based on impairment level such that, for those with high FMA-UE (>43, less impaired), IHI was stronger with greater impairment, while for those with low FMA-UE, stronger IHI was associated with better outcome in chronic stroke patients, suggesting the need for a stratified approach to neuromodulation [57]. Corticocortical reorganization with motor recovery also differs between recovery of simple vs. complex motor control. Interhemispheric connectivity between the two primary motor cortexes appears to correlate more with simple motor control, which is independent of CST integrity, suggesting a greater potential for recovery by contralesional vicariation of function as compared to complex motor skills, which depend on the integrity of the ipsilesional CST [58]. 

It is now becoming clear that NIBS should not be prescribed with a “one-size-fits-all” approach. Other than the factors listed above to consider the role of the contralesional hemisphere for NIBS, more studies are required to answer questions such as the optimal timing, site, and mode of stimulation as well as the type of stroke that would likely be responsive for NIBS. Task attributes including the limb segment involved, hand dominance, complexity of the task, and level of visual and sensory feedback should be given due consideration in future studies [8,49,59]. The STAC (Structural reserve, Task Attributes, Connectivity) approach has been proposed in the selection of NIBS approaches in relation to rehabilitation goals [60]. 

### 4.2. Possible Mechanisms by Which Facilitation of the Contralesional Hemisphere Improves Function

Mechanistically, the two most common NIBS methods differ in that tDCS delivers low-amplitude current through saline-soaked sponges over the scalp, modulating the resting membrane potentials of the neurons, whereas rTMS induces neuronal firing by the alternating the magnetic field from the coil. In either case, it is thought that NIBS modulates neuroplasticity through a myriad of effects on neurotransmission, local and distant cellular excitability, and potentially gene expression, although the exact mechanisms are still not fully known. 

Basic scientific studies supporting the facilitation of contralesional hemisphere show that in mice with middle cerebral artery occlusion, anodal tDCS to the contralesional prefrontal cortex (PFC) was found to yield better motor improvement compared to other tDCS montages, including anodal tDCS to ipsilesional PFC and bihemispheric stimulation [25]. In the same study, anodal tDCS to cM1 resulted in superior motor function compared to contralesional PFC and parietal tDCS. The cellular mechanisms of contralesional NIBS were attributed to higher expression of growth factors (growth/differentiation factor 5 and platelet-derived growth factor subunit A) and their receptors within the peri-infarct regions of the striatum. There were also higher numbers of proliferating neuronal cells in the lesioned sites, suggesting activation of intracellular cascade pathways for expression of growth factors in the lesioned hemisphere with contralesional facilitatory NIBS.

The human studies reviewed here suggest that functional improvement with contralesional facilitation is associated with increased contralesional cortical excitability [36] as well as variably with lesioned M1 excitability [39]. Functional effects may be mediated directly or indirectly, possibly through modulation of IHI [37,38] or via upregulation of uncrossed and extrapyramidal tracts [39,40]. 

Neurophysiologically, cPMD facilitation of iM1 is supported by studies using paired-coil TMS, which show greater cPMD-iM1 facilitation/lesser inhibition with greater impairment of arm function [61,62]. Studies in intracortical interactions with traditional stimulation paradigms, using paired-pulse TMS, have suggested a role for the disinhibition of GABAergic activity, both in the iM1 and cM1, associated with functional improvement [63,64]. 

However, failure-to-release IHI is noted to only emerge in the chronic phase of stroke as function improves, the degree of which correlates with the degree of residual impairment, contradicting the role of IHI in recovery and possibly suggesting the role of alternative contralesional corticoreticular projections in motor recovery [23].

Unmasking of latent ipsilateral CST pathways has been suggested as a possible mechanism by which contralesional facilitation affects motor recovery. This has been demonstrated in animal and human studies but may only be applicable in neurological lesions acquired in early life or the perinatal period [65,66,67,68]. Modulation of corticoreticulospinal or corticoreticulopropriospinal activity has been demonstrated with functional improvement following contralesional facilitation. These tracts from the primary, premotor, and supplementary areas project to the reticular formation, caudal pontine nuclei, and ipsilateral spinal cord via alpha motor neurons or propriospinal neurons [69,70].

### 4.3. Predictors of Response to Contralesional Hemispheric Facilitation

**Figure 2:**
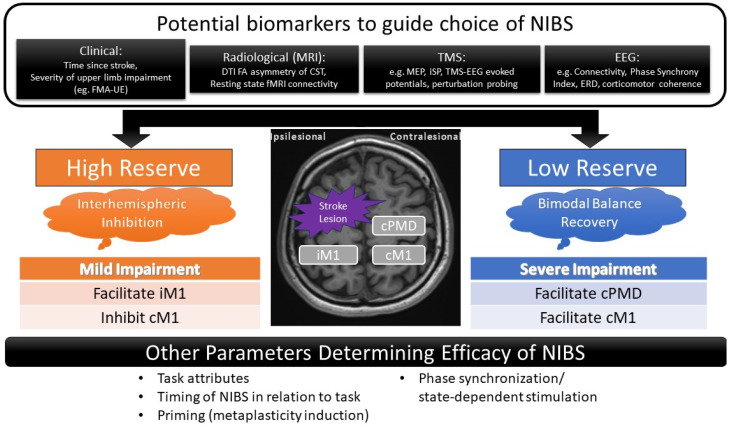
Selected possible biomarkers in choosing which NIBS protocols to use, accounting for severity of stroke (FM-UE: Fugl–Meyer Assessment for the Upper Extremity) and integrity of corticospinal tract (PLIC FA: posterior limb of internal capsule fractional anisotropy). iM1 = ipsilesional motor cortex; cM1 = contralesional motor cortex; cPMD = contralesional dorsal premotor cortex; iPMD = ipsilesional dorsal premotor cortex; DTI = Diffusion Tensor Imaging; FA = Fractional Anisotropy; FMA-UE = Fugl-Meyer Assessment–Upper Extremity; CST = Corticospinal Tract; ERD = Event Related Desynchronization; MEP = Motor Evoked Potential; iSP = Ipsilateral Silent Period.

The studies in this review suggest parameters that may help in the selection of the optimal NIBS approach and in predicting responses to contralesional facilitation (Figure 2). Sankarasubramaniam et al. proposed an algorithm based on FMA-UE score and MRI fractional anisotropy (FA) [37]. This is in addition to factors that are important in contralesional inhibition such as upper limb spasticity, functional impairment, as well as fractional anisotropy asymmetry on MRI and chronicity of stroke, which were associated with differential outcomes. 

In addition to being a prognostic biomarker, electrophysiological parameters may also be considered as predictors of response to NIBS. Measurements of IHI such as the ipsilateral silent period ratio (iSP ratio [71]), recorded when transcranial magnetic stimulation is applied over cM1, is one such biomarker. Subjects with a higher iSP ratio (indicating a higher degree of IHI from contralesional to ipsilesional hemisphere) may be more likely to respond to iM1 facilitation than cPMD facilitation [38]. By this same token, iSP ratio can be explored as a marker for cM1 facilitation [72]. 

Electroencephalography (EEG)-derived biomarkers may also be harnessed to determine the optimal site of stimulation with NIBS [73]. Intrahemispheric-phase synchrony index in the theta bands of cM1 correlated with motor gains, especially in more severely impaired patients [74]. Stronger contralesional event-related desynchronization (ERD) possibly effectuated by the mirror neuron system is seen in severely impaired patients; thus, changes in laterality coefficient may be explored as a therapeutic biomarker for NIBS [75]. Corticomotor coherence of the contralesional hemisphere in severely impaired patients during handgrip can be an adjunctive readout of recovery [76] and can be used potentially to stratify rTMS treatment paradigms [77]. Combined TMS-EEG studies of evoked potentials may shed light on intra- and intercortical interactions to inform individualized NIBS protocols [78]. Taken together, a closed loop system employing ERD to guide TMS stimulation may make the idea of a brain-state dependent stimulation a reality [79,80].

After stroke there is disruption of connectivity within and across hemispheres that is related to the degree of motor impairment and subsequent recovery [81]. For patients with severe CST damage, connectivity of cPMD with iM1 could be assessed to guide NIBS [82]. However, more studies are needed to directly investigate the effect of contralesional facilitatory NIBS on brain connectivity.

As our understanding of the effects of combined techniques such as TMS-TMS, TMS-EEG, and TMS-fMRI matures [83], perturbation probing may also be explored to optimize NIBS approaches [82]. A slowing of motor response to interference of cPMD or cM1 with TMS may be considered to identify patients who may respond to contralesional facilitation [35,54,56,84]. While some studies found that perturbation of either cPMD and cM1 impairs movement time, with no difference between sites [54], others found that perturbation of cPMD but not cM1 impaired movement time in the severe- but not in the mild-stroke group [54,85].

We summarize the relevant factors that may influence the choice between various NIBS protocols in Figure 2.

### 4.4. Study Limitations and Future Directions

The studies included were largely proof-of-concept studies with small sample sizes, and as a result, we could only conclude that contralesional facilitatory NIBS is feasible at this stage. The stimulation protocols used and the characteristics of stroke lesions sustained by the subjects in the trials included were also heterogenous. Therefore, it was not possible to conclude which subgroup would be more likely to benefit from the alternative contralesional facilitation approach. The sensitivity of outcome measures may differ based on stroke severity and choice of stimulated hemisphere: Movement kinematics improved more in severe stroke patients stimulated with atDCS contralesionally, suggesting that the way we measure NIBS outcomes may depend on the choice of ipsilesional vs. contralesional stimulation [86]. We might also have missed studies published in other languages or studies that were not indexed within the three databases in our search. One non-indexed study showed a positive effect of cPMD facilitation with non-navigated rTMS for proximal upper limb function [87]. 

Spasticity, a common post-stroke complication that negatively affects upper limb function, was only documented in three out of the eight studies, and one study excluded participants with significant spasticity [36]. We note that one study documented a trend of improvement of spasticity with contralesional facilitatory NIBS [39]. It is possible that some motor benefits seen with NIBS may be mediated through effects on spasticity [88], and future studies should assess the interactions between NIBS, spasticity, and motor function.

The optimal timing of NIBS utilization post stroke is yet another area of study. In animal studies, anodal tDCS application in the hyper-acute phase induced excitotoxic damage to the perilesional area [89] but, if applied later (after one week), resulted in improved function [90]. The brain is more excitable and capable of plastic changes in the first few months of stroke than in the later, more chronic phases [91]; thus, application of NIBS in the subacute phase might theoretically be more beneficial [92]. While a meta-analysis did not show differences in the efficacy of “early” vs. “late” NIBS [10], future studies directly addressing this issue would be required.

NIBS is considered a safe intervention, with a small risk of inducing seizures [93,94]. Published safety and operational guidelines exist for both rTMS and tDCS [93,95]. As this field moves forward, trials directly comparing the effects of rTMS and tDCS would be required. Technically, cPMD localization requires more precise navigation, as evidenced by Sankarasubramanian et al.’s findings that cM1 and cPMD were separated by 2.51 ± 1.01 cm. While tDCS of the premotor cortex has been attempted with smaller (3.5 × 5 cm) electrodes [96], more studies are required as to whether the spatial resolution of tDCS could be sufficient to target these sites.

With respect to differential effects of NIBS on different stroke subtypes, analysis of a large retrospective cohort applying LF-rTMS for upper limb rehabilitation to participants with chronic stroke found no difference between ischemic and hemorrhagic strokes [97]. Considering that hemorrhagic stroke accounts for a smaller proportion of all strokes, prospective studies will be required to answer whether this factor might affect the effectiveness of NIBS.

Finally, other stimulation targets of NIBS may need to be explored apart from cPMD and cM1, including but not limited to remote sites such as the contralesional anterior intraparietal sulcus [56] and cerebellum [98], as these may influence post-stroke motor recovery in ways that we are just beginning to understand. More studies are required to determine the role of these sites in relation to the timing post stroke.

## 5. Summary

This review provides preliminary evidence supporting facilitatory NIBS to the contralesional hemisphere to improve upper limb motor function after severe stroke. More studies are needed to investigate methods of stratification to determine NIBS approach, using a combination of clinical, MRI, and neurophysiological parameters. Task attributes and perturbation probing may also be useful in future studies. In an integrated rehabilitation program, NIBS should be positioned as an adjunct to therapy, which should be optimized for maximum effectiveness [8].

## Supplementary Materials

The following supporting information can be downloaded at: https://www.mdpi.com/article/10.3390/jcm13154420/s1, Table S1: Detailed Description of the 537 Studies Screened.

## Appendix A

Search strategy:

PubMed:

((((“ipsilateral”[All Fields] OR “ipsilaterality”[All Fields] OR “ipsilaterally”[All Fields] OR (“contralesional”[All Fields] OR “contralesionally”[All Fields]) OR “non-lesioned”[All Fields] OR (“unaffected”[All Fields] OR “unaffecteds”[All Fields])) AND (“high-frequency”[All Fields] OR “anodal”[All Fields] OR (“intermittant”[All Fields] OR “intermittence”[All Fields] OR “intermittencies”[All Fields] OR “intermittency”[All Fields] OR “intermittent”[All Fields] OR “intermittently”[All Fields]) OR (“facilitate”[All Fields] OR “facilitated”[All Fields] OR “facilitates”[All Fields] OR “facilitating”[All Fields] OR “facilitation”[All Fields] OR “facilitations”[All Fields] OR “facilitative”[All Fields] OR “facilitator”[All Fields] OR “facilitator s”[All Fields] OR “facilitators”[All Fields]) OR (“activable”[All Fields] OR “activate”[All Fields] OR “activated”[All Fields] OR “activates”[All Fields] OR “activating”[All Fields] OR “activation”[All Fields] OR “activations”[All Fields] OR “activator”[All Fields] OR “activator s”[All Fields] OR “activators”[All Fields] OR “active”[All Fields] OR “actived”[All Fields] OR “actively”[All Fields] OR “actives”[All Fields] OR “activities”[All Fields] OR “activity s”[All Fields] OR “activitys”[All Fields] OR “motor activity”[MeSH Terms] OR (“motor”[All Fields] AND “activity”[All Fields]) OR “motor activity”[All Fields] OR “activity”[All Fields])) AND (“transcranial magnetic stimulation”[MeSH Terms] OR (“transcranial”[All Fields] AND “magnetic”[All Fields] AND “stimulation”[All Fields]) OR “transcranial magnetic stimulation”[All Fields] OR (“repetitive”[All Fields] AND “transcranial”[All Fields] AND “magnetic”[All Fields] AND “stimulation”[All Fields]) OR “repetitive transcranial magnetic stimulation”[All Fields] OR “transcranial magnetic stimulation”[MeSH Terms] OR (“transcranial direct current stimulation”[MeSH Terms] OR (“transcranial”[All Fields] AND “direct”[All Fields] AND “current”[All Fields] AND “stimulation”[All Fields]) OR “transcranial direct current stimulation”[All Fields]) OR “transcranial direct current stimulation”[MeSH Terms] OR ((“theta”[All Fields] OR “thetas”[All Fields]) AND (“burst”[All Fields] OR “bursting”[All Fields] OR “bursts”[All Fields]) AND (“stimulate”[All Fields] OR “stimulated”[All Fields] OR “stimulates”[All Fields] OR “stimulating”[All Fields] OR “stimulation”[All Fields] OR “stimulations”[All Fields] OR “stimulative”[All Fields] OR “stimulator”[All Fields] OR “stimulator s”[All Fields] OR “stimulators”[All Fields])) OR (“non-invasive”[All Fields] AND (“brain stimul”[Journal] OR (“brain”[All Fields] AND “stimulation”[All Fields]) OR “brain stimulation”[All Fields]))) AND (“stroke”[MeSH Terms] OR “stroke”[All Fields] OR “strokes”[All Fields] OR “stroke s”[All Fields] OR “stroke”[MeSH Terms] OR (“stroke”[MeSH Terms] OR “stroke”[All Fields] OR (“cerebrovascular”[All Fields] AND “accident”[All Fields]) OR “cerebrovascular accident”[All Fields]) OR “stroke”[MeSH Terms]) AND (“motor”[All Fields] OR “motor s”[All Fields] OR “motoric”[All Fields] OR “motorically”[All Fields] OR “motorics”[All Fields] OR “motoring”[All Fields] OR “motorisation”[All Fields] OR “motorised”[All Fields] OR “motorization”[All Fields] OR “motorized”[All Fields] OR “motors”[All Fields])

Scopus:

(((anodal)) OR ((high-frequency)) OR ((intermittent)) OR ((facilitation)) OR ((activation))) AND (((ipsilateral)) OR ((contralesional)) OR ((unaffected)) OR ((non-lesioned))) AND (((repetitive AND transcranial AND magnetic AND stimulation)) OR (transcranial AND direct AND current AND stimulation)) OR ((non-invasive AND brain AND stimulation)) OR ((theta AND burst AND stimulation))) AND (((cerebrovascular AND accident)) OR ((stroke))) AND ((motor))

Web of Science:

[stroke (All Fields) OR cerebrovascular accident (All Fields)] AND [repetitive transcranial magnetic stimulation (All Fields) OR transcranial direct current stimulation (All Fields) OR theta burst stimulation (All Fields) OR non-invasive brain stimulation (All Fields)] AND motor (All Fields)] AND [contralesional (All Fields) OR non-lesioned (All Fields) OR unaffected (All Fields) OR ipsilateral (All Fields)] AND [high-frequency (All Fields) OR anodal (All Fields) OR facilitation (All Fields) OR activation (All Fields) OR intermittent (All Fields)]
